# Dopamine-loaded nanoparticle systems circumvent the blood–brain barrier restoring motor function in mouse model for Parkinson’s Disease

**DOI:** 10.1038/s41598-021-94175-8

**Published:** 2021-07-26

**Authors:** Victoria Monge-Fuentes, Andréia Biolchi Mayer, Marcos Robalinho Lima, Luiza Ribeiro Geraldes, Larissa Nepomuceno Zanotto, Karla Graziella Moreira, Olimpia Paschoal Martins, Henrique Luís Piva, Maria Sueli Soares Felipe, Andre Correa Amaral, Anamélia Lorenzetti Bocca, Antonio Claudio Tedesco, Márcia Renata Mortari

**Affiliations:** 1 Laboratório de Neurofarmacologia, Departamento de Ciências Fisiológicas, Instituto de Ciências Biológicas, Universidade de Brasília, Brasília, 70910-900 Brazil; 2 Departamento de Química, Centro de Nanotecnologia e Engenharia de Tecidos-Fotobiologia e Fotomedicina, Faculdade de Filosofia, Ciências e Letras de Ribeirão Preto, Universidade de São Paulo, Av. Bandeirantes 3900, Ribeirão Preto, SP 14040-901 Brazil; 3 Laboratório de Imunologia Aplicada, Departamento de Biologia Celular, Instituto de Ciências Fisiológicas, Universidade de Brasília, Brasília, 70910-900 Brazil; 4 Departamento de Biologia Animal e Plantas, Centro de Ciências Biológicas, Universidade Estadual de Londrina, Londrina, 86051-970 Brazil; 5 Laboratório de Fisiologia e Farmacologia, Universidade Federal de Catalão, Goiás, 75704-020 Brazil; 6 Universidade Católica de of Brasília, Campus Asa Norte, Asa Norte, Brasília, 70790-160 Brazil; 7 Instituto de Saúde Pública e Patologia Tropical, Universidade Federal de Goiás, Goiânia, Goías Brazil

**Keywords:** Drug discovery, Neuroscience, Diseases, Nanoscience and technology

## Abstract

Parkinson's disease (PD) is a progressive and chronic neurodegenerative disease of the central nervous system. Early treatment for PD is efficient; however, long-term systemic medication commonly leads to deleterious side-effects. Strategies that enable more selective drug delivery to the brain using smaller dosages, while crossing the complex brain-blood barrier (BBB), are highly desirable to ensure treatment efficacy and decrease/avoid unwanted outcomes. Our goal was to design and test the neurotherapeutic potential of a forefront nanoparticle-based technology composed of albumin/PLGA nanosystems loaded with dopamine (ALNP-DA) in 6-OHDA PD mice model. ALNP-DA effectively crossed the BBB, replenishing dopamine at the nigrostriatal pathway, resulting in significant motor symptom improvement when compared to Lesioned and L-DOPA groups. Notably, ALNP-DA (20 mg/animal dose) additionally up-regulated and restored motor coordination, balance, and sensorimotor performance to non-lesioned (Sham) animal level. Overall, ALNPs represent an innovative, non-invasive nano-therapeutical strategy for PD, considering its efficacy to circumvent the BBB and ultimately deliver the drug of interest to the brain.

## Introduction

Parkinson's disease (PD) is a progressive, complex, and chronic neurodegenerative disorder/syndrome associated with an array of cardinal motor manifestations (bradykinesia, postural instability, muscle stiffness, resting tremor, and rigidity)^1,2^. PD is often accompanied by debilitating non-motor symptoms that include olfactory dysfunction, sleep disturbances, gastrointestinal disorders, genitourinary dysfunctions, pain, and neuropsychiatric symptoms^3,4^. As the disease chronically progresses with time, symptoms tend to worsen, deeply affecting the patients’ quality of life.

Neuropathological hallmarks of PD frequently include the degeneration of dopamine (DA) neurons in the *substantia nigra pars compacta* (SNpc) with consequent striatal DA deficiency, coupled with cytoplasmic proteinaceous aggregates in nerve cells and terminals, structures referred to as Lewy bodies and Lewy neurites, respectively^5,6^. As a result, the progressive and continuous destruction of dopaminergic neurons at the nigrostriatal pathway jeopardizes the production, storage, and release of dopamine, gradually leading to the characteristic loss of motion control and coordination. PD additionally involves deficits in multiple non-dopaminergic neurotransmitter pathways including the cholinergic, serotonergic and noradrenergic systems, having an impact on a range of neuronal networks and brain regions^2,7,8^, accounting for a series of non-motor symptoms.

Although PD was first described more than 200 years ago^9^, disease-modifying drugs are unavailable, thus, patients' relief is sought through symptomatic improvement by compensating decreasing nigrostriatal dopaminergic levels, offered by frontline medications (dopamine agonists ropinirole, rotigotine, bromociptine, and pramipexole) and non-pharmacological methods (deep brain stimulation, MRI-guided focused ultrasound, rehabilitative therapy, and exercise)^10^. In the case of more advanced PD patients, a dopamine replacement strategy has been adopted as the gold-standard since the late 1960s, through the use of a dopamine precursor known as Levodopa or L-DOPA (L-3,4-dihydroxyphenylalanine)^11^. L-DOPA can be used as monotherapy or in combination with add-on non-dopaminergic drugs, benefiting PD patients with significant and dramatic symptomatic relief^12^. However, after 5–10 years of chronic L-DOPA therapy, several side effects (dyskinesia, motor fluctuations, neuropsychiatric complications, sleep disturbances, non-motor fluctuation, *on–off* effects, and wearing-off effect) are detected in most patients^13^, reaching a point where the side effects are greater than the therapeutic benefits. Therefore, considering the limitations of current PD therapies and the alarming prevalence projections for the oncoming decades, the development of adjuvant and innovative technologies becomes imperative to detect, slow or even halt the progression of the disease.

Finding new pharmaceutical forms that ultimately facilitate the transport of already well-established medications across the complex structure of the blood–brain barrier (BBB) is imperative to efficaciously deliver treatment to the brain tissue^14^. Among the various available alternatives, nanoparticle-based technology is positioned as a forefront approach for disease treatment considering the myriad of nanomaterials that grant positive features for theranostic drug delivery (DD) strategies^15,16^. Particularly for neurodegenerative disease treatment, the design of biodegradable polymeric nanoparticles (NP) with appropriate surface modifications allow drug delivery to the brain by targeting/crossing the BBB via receptor-mediated pathways. This DD strategy is further attractive considering that BBB integrity and normal functioning are preserved, while delivering drug payloads to the brain, including BBB-impermeable therapeutic agents^17^, such as dopamine. Considering that PD treatment by dopamine (DA) replenishment is hindered by this drug's hydrophilic nature, an unfavorable characteristic for BBB crossing^18^, the formulation of dopamine-loaded nanoparticles has been explored to modulate treatment bioavailability, efficacy, and stability. Several types of nanomaterials, such as liposomes^19^, chitosan NPs^20,21^, quantum rods^22^, cellulose acetate phthalate (CAP)^23^, borneol and lactoferrin co-modified NPs^24^, and solid lipid nanoparticles^25^ have been synthesized to encapsulate DA. Some of these nanomaterial-based technologies have already been tested in PD pre-clinical models; however, to the best of our knowledge, no studies have been completed for the treatment of PD at the clinical level^26^.

Dopamine has also been incorporated into poly (D,L-lactic-*co*-glycolic acid) (PLGA) polymeric nanoparticles, a material that confers optimized metabolism, stability, increased bioavailability, and biocompatibility. Treatment with dopamine-loaded PLGA NPs ultimately aims to improve physical endurance, coordination, and anxiety in animal models^12,17,27^. Based on PLGA’s pharmacokinetic/pharmacodynamic advantages and our intention to enhance drug delivery to the brain, albumin was further selected for the synthesis of our experimental nanosystems (ALNP), mainly considering the compound's ability to permeate the BBB via receptor-mediated pathways^28^. In our initial experiments, ALNPs’ composition included fluorophore marker aluminum chloride phthalocyanine (AlClPc) to track nanosystem passage through the BBB. AlClPc offers convenient characteristics for a photoactive compound, for instance, strong absorption of visible light, high affinity and selective penetration into biological tissue, favorable pharmacokinetics, high stability, and low drug toxicity in the absence of visible light used in photoactivation^29^.

Herein we report the preparation of nanosystems with special composition and properties aimed to enhance dopamine delivery at the brain tissue by circumventing the complex BBB structure. This strategy emerges as a promising route to target drugs to the brain and as a useful photodiagnostic tool of the region by visible light detection. We further tested the potential of these nanoparticles to restore neurobehavioral and neurochemical deficits in hemiparkinsonian mice using the 6-hydroxydopamine (6-OHDA) model that causes the selective death of dopaminergic neurons.

## Results

### Nanoparticle preparation and characterization

**Figure 1:**
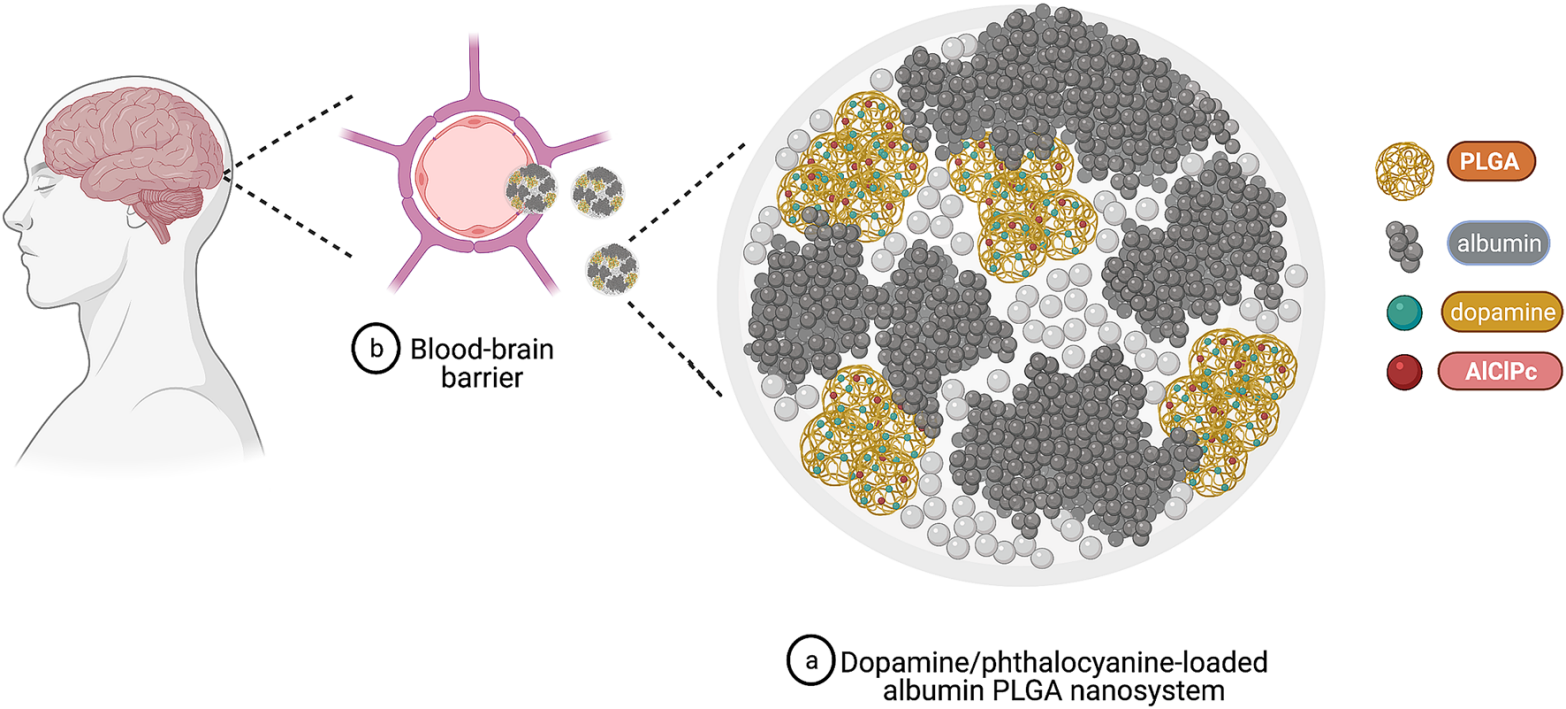
Schematic representation of our dopamine/phthalocyanine-loaded nanosystem’s structural assembly (**a**) and its interaction with the blood–brain barrier (**b**) to ultimately enter the brain parenchyma and release the drug/compound of interest. *AlClPc* aluminum chloride phthalocyanine, *PLGA* poly (lactic-*co*-glycolic acid). Created with Biorender.com.

**Figure 2:**
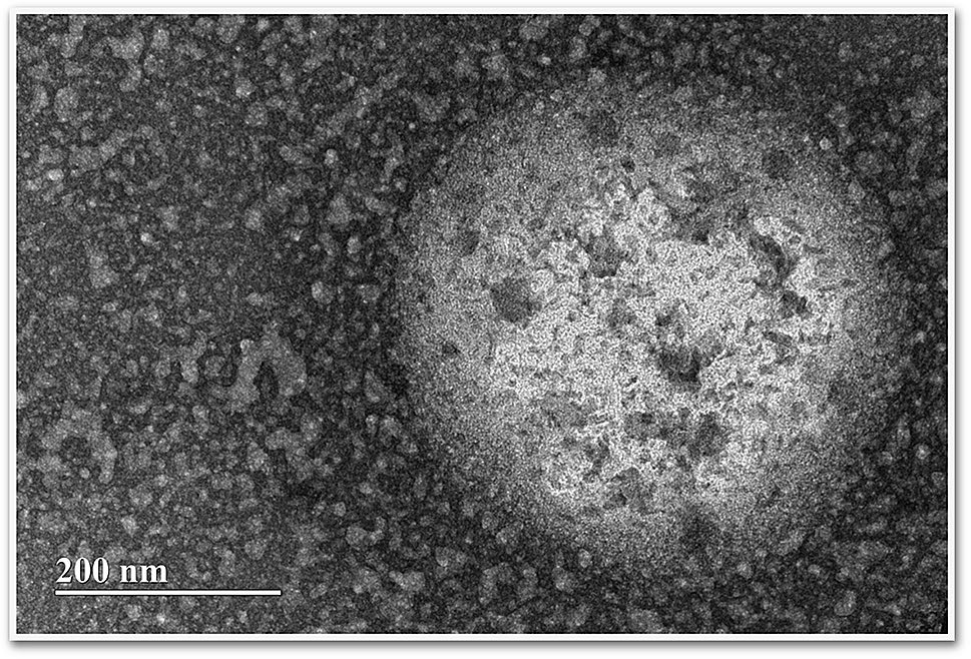
Nanosystem characterization. Transmission electron micrograph of a representative albumin/PLGA nanosystem, in this case, depicting a phthalocyanine-loaded albumin/PLGA (ALNP + AlClPc) nanosystem at 30,000× magnification showing spherical format and size of 466 nm. Scale bar: 200 nm.

Our rationale was to synthesize a nanosystem with specific composition and physicochemical properties that facilitate BBB crossing, enhancing dopamine delivery to the brain tissue (Fig. 1). Thus, we first examined the physicochemical properties of all albumin/PLGA nanosystems formulated (Table 1). Analysis revealed polydispersity index (PDI) results that varied from 0.4 to 0.6, indicating a narrow size distribution. Regarding zeta potential, nanosystems presented a negative surface charge (− 27 to − 37 mV). With respect to hydrodynamic diameter, values varied from 353 to 497 nm. Noteworthy, a considerable increase of around 46% in mean particle size was observed for ALNPs functionalized with aluminum chloride phthalocyanine (497 nm) when compared to unloaded-ALNPs (340 nm). Despite the mentioned size increase, DDS capacity to reach the brain parenchyma and deliver AlClPc was not affected. ALNP + AlClPc nanosystem analysis by TEM revealed spherical morphology (Fig. 2) and average diameter of 466 nm, in line with the obtained DLS size result.

**Table 1:** Physicochemical nanoparticle characterization.

Formulation	HD (nm)	PDI	ζ-potential (mV)
Unloaded ALNP	340	0.4	− 27
ALNP + DA	353	0.5	− 37
ALNP + AlClPc	497	0.6	− 29

Physicochemical parameters observed in the different sub-variations of albumin/PLGA nanosystems tested, as determined by dynamic light scattering.

Values were determined as the mean of three runs (10 measurements/run).

*ALNP* albumin/PLGA nanosystems, *AlClPc* aluminum chloride phthalocyanine, *DA* dopamine, *HD* hydrodynamic diameter, *PDI* polydispersity index, *ζ-potential* zeta potential.

### Nanoparticle delivery across the BBB

**Figure 3:**
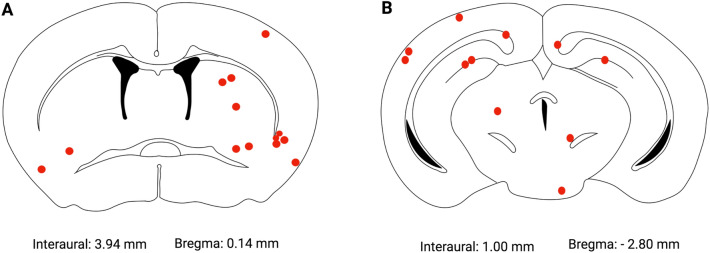
Representation of nanoparticle agglomerates (red dots) localization in mice brain treated with nanosystems functionalized with traceable fluorophore aluminum chloride phthalocyanine (ALNP + AlClPc 20 mg); (**A**) Striatal region. (**B**) Hippocampal formation region (30). *N* = 5–7.

**Figure 4:**
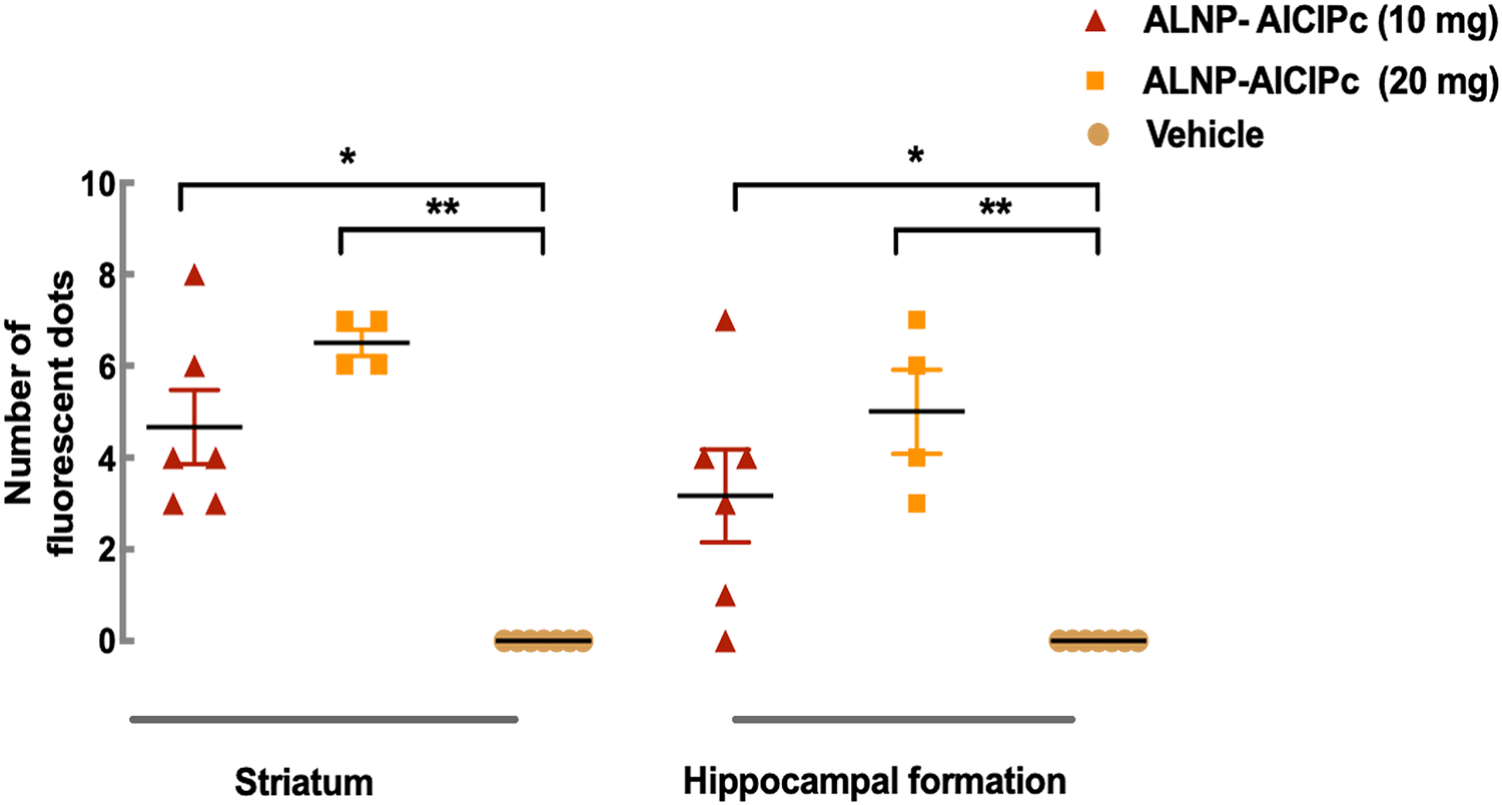
Quantification of fluorescent nanoparticle agglomerates (fluorescent dots) observed at the striatum or hippocampal formation according to treatment groups (saline vehicle or albumin/PLGA nanosystems functionalized with photoactive compound aluminum chloride phthalocyanine, ALNP + AlClPc, at concentrations of 10 or 20 mg/animal), 1 h post-injection. Data were submitted to Kruskal–Wallis test, followed by Dunn post-test. (*) *p* < 0.05 and (**) *p* < 0.001 when compared to vehicle group. *N* = 5–7.

We next evaluated if our albumin/PLGA nanosystem (photolabed with AlClPc) presented suitable characteristics to circumvent the BBB and, thus, be found in the brain tissue. Analysis revealed red fluorescent dots scattered throughout the tissue indicating the presence of clusters/agglomerates of nanoparticles that emit light at 680 nm. Our albumin/PLGA nanosystem agglomerates were located throughout the brain, being also detected at the hippocampal formation and striatum (Fig. 3). While comparing slides from both regions, the striatum showed a greater amount of NP agglomerates [Kruskal–Wallis statistic = 26.35, *p* < 0.0001], for both ALNP + AlClPc (10 and 20 mg/animal) doses. Notably, control samples did not present any fluorescent dots, and only the cell nuclei labeled with blue fluorescent DAPI were visualized. Analyzed groups treated with ALNP + AlClPc (10 mg and 20 mg/animal) were significantly different when compared to the vehicle group (*p* < 0.01 to both groups in relation to ALNP + AlClPc 10 mg/animal and *p* < 0.05 to ALNP + AlClPc 20 mg/animal) (Fig. 4).

### Dopamine-loaded nanoparticle treatment efficacy

Once the presence of fluorescent albumin/PLGA + AlClPc nanoparticles in the brain parenchyma was confirmed, an effective dopamine dose to be delivered by our nanosystem was determined. Thereafter, the therapeutic potential of our dopamine-loaded delivery systems was evaluated by performing neurobehavioral tests to assess motor coordination, balance, sensorimotor function, and overall dopamine responsiveness.

**Figure 5:**
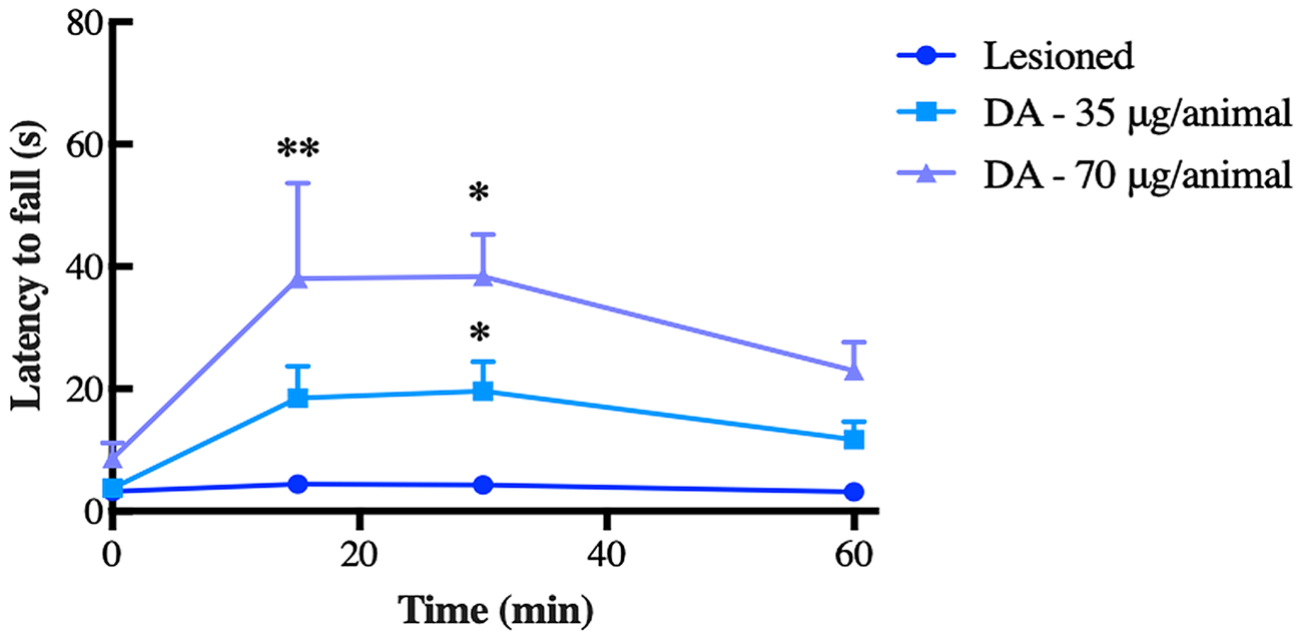
Latency to fall (*s*) from the rotarod in 6-OHDA-lesioned mice treated with saline or bulk dopamine (DA) either at 35 or 70 µg/animal. Data were submitted to ANOVA multivariate analysis, followed by Tukey post-hoc test. Significantly different from lesioned group, (*) *p* < 0.05 and (**) *p* < 0.01. *N* = 7.

Lesioned animals treated i.c.v. with bulk dopamine at a dose of 70 µg/animal showed significant longer latency periods when compared to the other treatments, specially during the first 30 min of test (*p* < 0.01 to 15 min and *p* < 0.05 to 30 min in relation to untreated-Lesioned group), suggesting the efficacy of this dose to improve balance and motor coordination (Fig. 5). Two-way ANOVA showed significant results for time [F_(2,28)_ = 8.78, p < 0.01], treatment [F_(2,18)_ = 6.50, *p* < 0.01], and time-treatment interaction [F_(6,54)_ = 2.35, p < 0.05]. Thereafter, the dose of 70 µg of dopamine in 10 mg of ALNP was used for our neurobehavioral assays, along with the dose of 140 µg of dopamine per 20 mg of ALNP (7 µg of dopamine/mg of nanoparticles).

### Rotarod test

**Figure 6:**
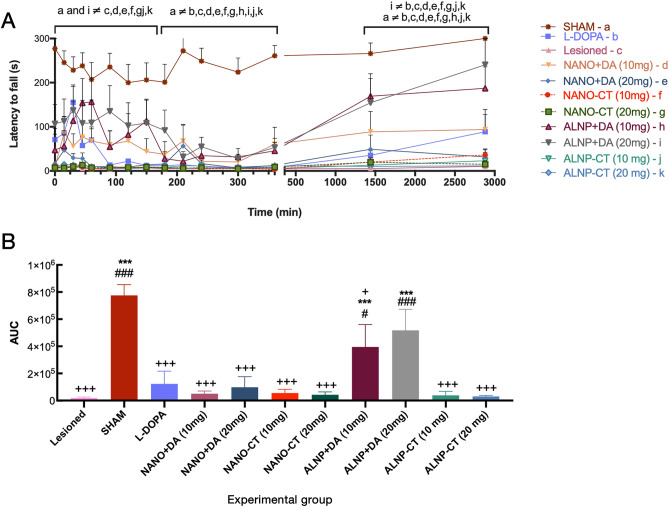
(**A**) Latency to fall (*s*) from the rotarod in Sham and 6-OHDA-lesioned mice treated either with bulk L-DOPA, NANO-Control (NANO-CT; 10 or 20 mg/animal; NANO + Dopamine (NANO-DA; 10 or 20 mg/animal); dopamine-loaded-albumin/PLGA nanosystems (ALNP + DA; 10 or 20 mg/animal); and unloaded albumin/PLGA nanosystems (ALNP-CT; 10 and 20 mg/animal). Data were submitted to ANOVA multivariate analysis, followed by the Bonferroni post-test. Letters indicate statistically significant difference (*p* < 0.001). (a) Sham, (b) L-DOPA, (c) Lesioned, (d) NANO + DA 10 mg, (e) NANO + DA 20 mg, (f) NANO-CT 10 mg, (g) NANO-CT 20 mg, (h) ALNP + DA 10 mg, (i) ALNP + DA 20 mg, (j) ALNP-CT 10 mg, (k) ALNP-CT 20 mg. *N* = 7 animals/group. (**B**) Area under the curve (AUC) analysis for the latency to fall test in Sham and 6-OHDA-lesioned mice submitted to the mentioned treatments in A. Data were submitted to one-way ANOVA, followed by Tukey post-test. Significant differences: (***) *p* < 0.001 in relation to Lesioned group; (^+^) *p* < and (^+++^) *p* < 0.001 in relation to Sham group; (^#^) *p* < 0.05 and (^###^) *p* < 0.001 in relation to L-DOPA group. *N* = 7 animals/group.

In 6-OHDA-lesioned animals, mice from group treated with ALNP + DA 20 mg/animal remained on the rotarod for a longer period of time, when compared to the other treatment groups [two-way ANOVA: time F_(14,98)_ = 10.81, p < 0.0001; treatment F_(10,70)_ = 14.82, p < 0.0001, and time-treatment interaction F_(140,980)_ = 2.684, p < 0.0001]. ALNP + DA 20 mg (in the period of 0 to 180 min, 1440 and 2880 min after administration) presented an overall better performance even when compared to group treated only with bulk L-DOPA, currently used as frontline treatment against PD [p < 0.001 in periods between 120 and 180 min, as well as 1440 min (24 h) and 2880 min (48 h) after treatment]. On the other hand, group treated with unloaded ALNP-CT did not present any symptomatic improvement and fall latency results showed no significant difference when compared to untreated 6-OHDA-lesioned animals (Fig. 6A). To further support our findings, additional data analysis using area under the curve (AUC) indicated significant differences [F_(10, 62)_ = 21.91, p < 0.0001] (Fig. 6B). Post-test revealed significant permanence improvement on the rotational behavior test for groups treated with ALNP + DA 10 mg (when compared to Lesioned *p* < 0.01; Sham *p* < 0.05; and L-DOPA *p* < 0.05) and ALNP + DA 20 mg (*p* < 0.001 for both Lesioned and L-DOPA) demonstrating potential antiparkinsonian activity. Noteworthy, Sham and ALNP + DA 20 mg rotational results did not show significant difference. When contrasting latency times for animals treated with NANO + DA or ALNP + DA at corresponding doses, we observed that ALNP + DA showed longer latency periods, supporting the above discussed role that albumin plays in facilitating nanoparticle passage through the BBB.

### Adhesive removal test

**Figure 7:**
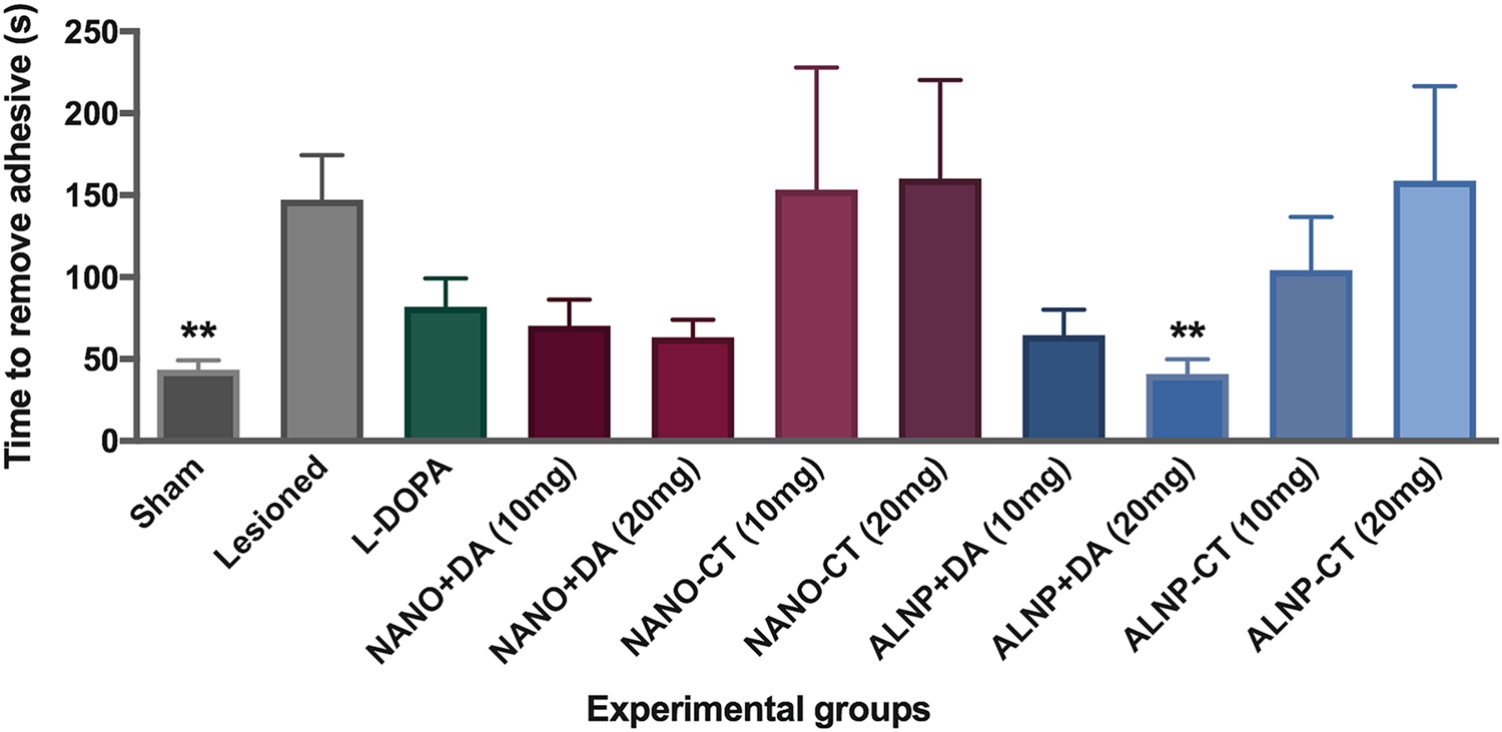
Latency to remove adhesive in Sham and 6-OHDA-lesioned animals treated with bulk L-DOPA, NANO-control (NANO-CT; 10 or 20 mg/animal; NANO + Dopamine (NANO + DA; 10 or 20 mg/animal); dopamine-loaded albumin/PLGA nanosystems (ALNP-DA; 10 or 20 mg/animal); unloaded albumin/PLGA nanosystems (ALNP-CT; 10 and 20 mg/animal). Data were submitted to one-way ANOVA analysis of variance, followed by the Tukey post-test. (**) Statistically significant difference in relation to the 6-OHDA group (*p* < 0.01). *N* = 7 animals/group.

The adhesive removal test indicated that animals treated with ALNP + DA 20 mg presented similar results to Sham control group, with respect to time to remove adhesive on the contralateral forepaw, with significant difference (*p* < 0.01) when contrasted to untreated 6-OHDA-lesioned animals (Fig. 7) [F_(10,75)_ = 3.745, *p* < 0.0004]. As observed, all unilateral 6-OHDA lesioned mice, except for those treated with ALNP + DA 20 mg, showed impairments in the adhesive removal test.

### Apomorphine-induced rotation test

**Figure 8:**
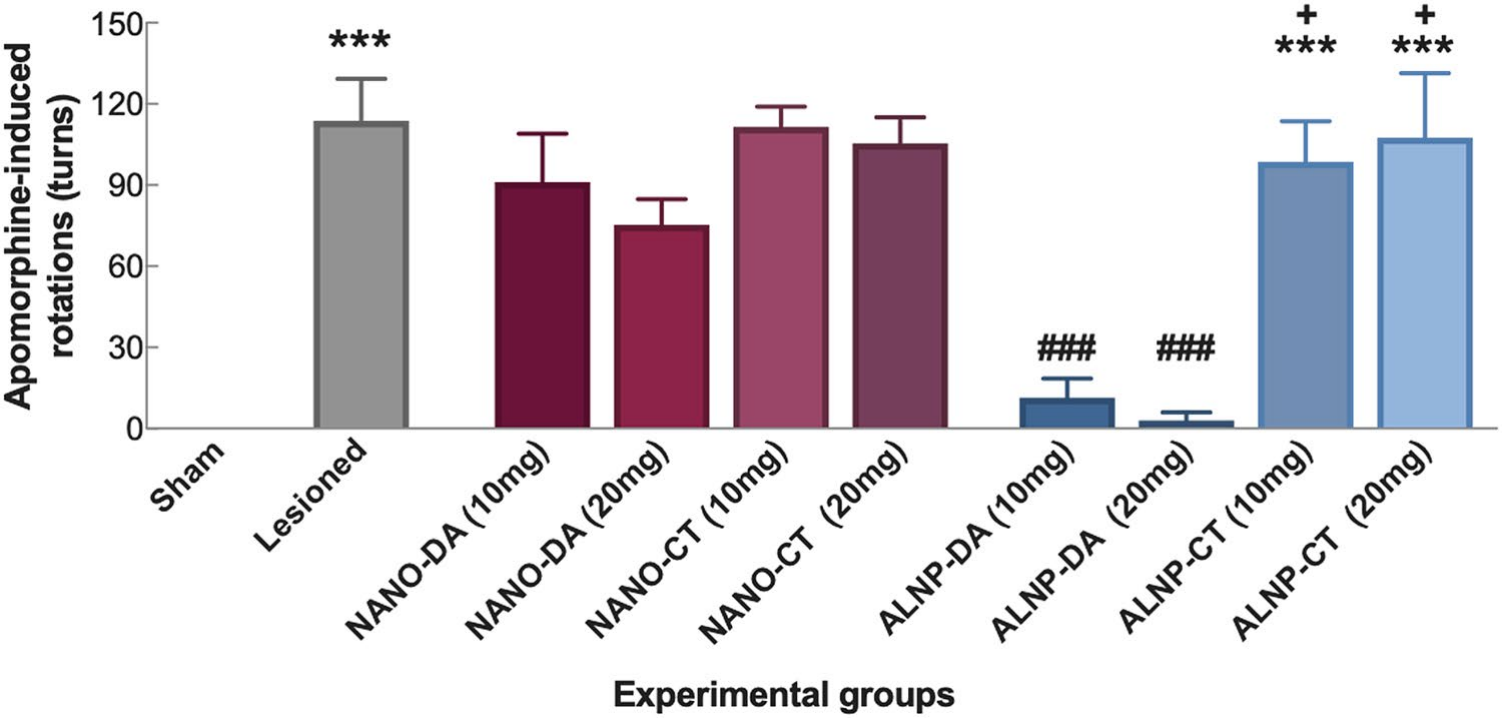
Apomorphine-induced contralateral rotations in Sham and 6-OHDA-Lesioned animals treated with NANO-Control (NANO-CT; 10 or 20 mg/animal; NANO + Dopamine (NANO + DA; 10 or 20 mg/animal); dopamine-loaded albumin/PLGA nanosystems (ALNP-DA; 10 or 20 mg/animal); and unloaded control albumin/PLGA nanosystems (ALNP-CT; 10 and 20 mg/animal). Data are expressed as number of contralateral turns, submitted to one-way ANOVA analysis, followed by the Tukey post-test. (***) *p* < 0.001 when compared to Sham group; (^###^) *p* < 0.001 with respect to Lesioned group; (^+^) *p* < 0.01 compared to ALNP-DA 20 mg. *N* = 7 animals/group.

One-way ANOVA revealed significant differences in the apomorphine-induced spontaneous rotation test [F_(8,40)_ = 13.53, p < 0.001] (Fig. 8). Assessment of these rotations showed that mice treated with either dose of ALNP + DA presented significant reduction in the number of apomorphine-induced rotations when compared to untreated Lesioned animals (*p* < 0.001). Additionally, significant difference was observed when mice treated with ALNP + DA 20 mg were contrasted with group treated with unloaded-albumin/PLGA nanosystems (ALNP-CT) (*p* < 0.01).

### TH^+^-immunohistochemistry

**Figure 9:**
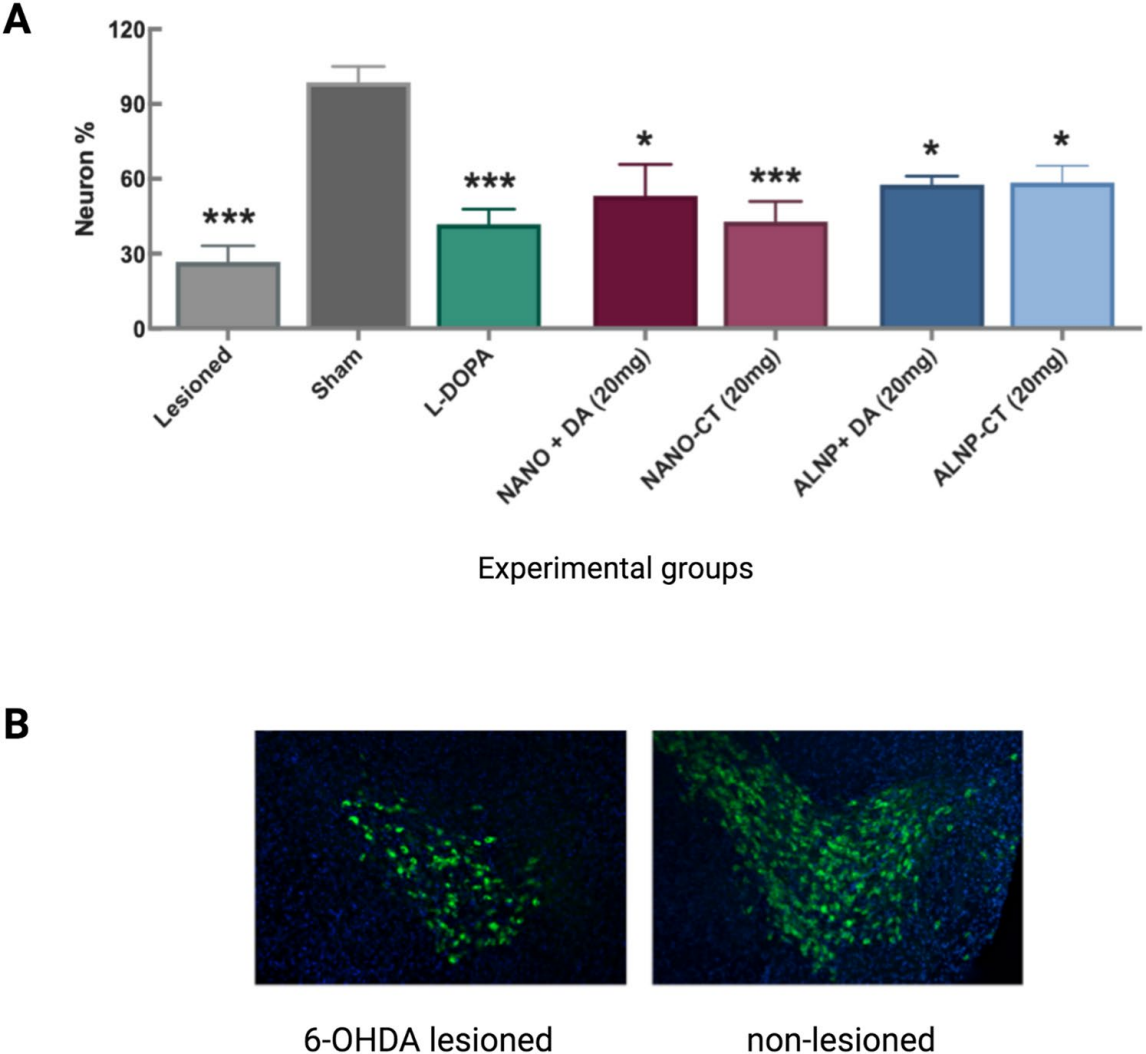
(**A**) Data show percentage of remaining dopaminergic neurons reactive to tyrosine hydroxylase (TH^+^) on the 6-OHDA lesioned side, compared to the intact hemisphere (corresponds to 100%), according to experimental group. Data were submitted to one-way ANOVA analysis of variance, followed by the Tukey post-test. Graphs show mean ± SEM. (*) *p* < 0.05 and (***) *p* < 0.001 when compared to Sham. (**B**) Fluorescence micrographs of *substantia nigra* (SN) coronal sections stained for TH^+^ show the non-lesioned (contralateral) and 6-OHDA lesioned (ipsilateral) SN, inducing partial TH^+^ cell loss. Animals were treated with dopamine-loaded albumin/PLGA nanosystems (ALNP-DA) (20 mg/animal). Magnification: ×10. *N* = 7 animals/group.

Analysis counted the number of neurons reactive to antibody tyrosine hydroxylase (TH^+^) at the *substantia nigra* (SN) and at the Ventral Tegmental Area (VTA) in both hemispheres (left ipsilateral: 6-OHDA lesioned, right contralateral: non-lesioned). The objective in mind was to examine the damage extent on DA neurons caused by the i.c.v. injection of 6-OHDA and verify if treatment with NANO and ALNP nanoparticles presented a neuroprotective effect. Our results confirmed that the 6-OHDA dosage used promoted tissue lesioning [F_(8,38)_ = 9.217, *p* < 0.0001] that resulted in a significant reduction of TH^+^ fibers (mean ± SEM: 73.4 ± 6.5% from the contralateral side), discernible from Sham (1.3 ± 6.4% of the contralateral side). Significant difference was observed only for Sham group when compared to the other treatment groups (Fig. 9A). TH^+^-immunolabeling of viable SN dopaminergic neurons revealed that independently from the treatment group, even for ALNP 20 mg that showed promising results in the neurobehavioral tests, the percentage of neurons from the lesioned side was reduced when compared to DA neurons at SN from the non-lesioned side (Fig. 9B).

## Discussion

Nanoparticle-based technologies have emerged as platforms able to carry and release drug payloads at the target site. This objective can be fulfilled by carefully choosing appropriate composition and physicochemical properties that assure nanoparticle biological behavior, successful cellular uptake, and drug delivery capacity while avoiding toxicity^30^.

As a starting point, a suitable carrier was selected, thus, an FDA-approved PLGA polymeric blend was chosen for the structural synthesis of all NPs tested, considering that it confers to the encapsulated drug several pharmacokinetic advantages such as more stability, clearance reduction, biocompatibility, biodegradability, and increased plasma half-life in comparison to the bulk drug^12,17,31^. As shown by Pahuja et al.^12^, DA entrapment in PLGA-based NPs further permits slow and sustained release, while protecting the drug from oxidative metabolism, ultimately decreasing cell toxicity. Other in vivo studies have also described the potential of PLGA nanoparticles for brain delivery as carriers for drugs and compounds such as curcumin^32^, loperamide^33^, dopamine^12^, methotrexate^34^, and paclitaxel^35^, intended for the treatment of brain tumors or neurodegenerative diseases.

Since our primordial goal was to enhance drug delivery to the brain, albumin was particularly selected to be part of our experimental nanosystems (ALNP) based on its capacity to permeate the BBB via receptor-mediated pathways, while granting biocompatibility, non-immunogenicity, and versatility as a drug carrier system^18,28^ solving solubility problems for hydrophilic drugs, such as dopamine. Albumin is further widely used to extend the half-life, enhance stability, avoid degradation and facilitate specific targeting of therapeutic agents^36^. Additionally, considering that albumin is a dysopsonin, its use as a surface modifier reduces the binding of other proteins, decreasing NP reticular endothelial uptake^37^.

In view of the physicochemical properties of our nanosystems, the observed narrow size distribution could be attributed to the use of PLGA, a polymer that possesses high viscosity composition (polymer ratio 50:50) and better emulsifying properties given by its higher molecular weight (MW 100,000 Da), ultimately leading to more uniform particle size distribution^38^. The negative surface charge obtained represents a favorable characteristic for NP passage through the BBB, which further avoids toxicity by cell wall disruption^39,40^.

Concerning particle size range, literature reports that nanocarriers that favor drug deposition at the brain tissue should vary between 50 and 200 nm^41^. Notably, these studies take into account only the dry and “bare" nanoparticles with a size depicted only by the area of the core, which do not truly exist in biological fluids, where they become hydrated and modified by proteins that accumulate on their surfaces forming a corona that increases the particles’ hydrodynamic diameter^42^. Based on our results for the BBB passage assay and neurobehavioral treatment efficacy, the HD obtained, ranging from 353 to 497 nm, was suitable for NPs to reach the brain parenchyma and deliver dopamine or AlClPc, as observed by symptom improvement or fluorescence detection, respectively. Furthermore, the HD augmentation seen in ALNPs functionalized with AlClPc might be attributed to particular interactions between AlClPc molecules and albumin proteins during DDS formulation. This process might mimick classical aggregation processes related to physicochemical factors previously described for phtalocyanines^43^. On this sense, aluminum phthalocyanines exhibit a high affinity binding site constant (K ~ 3–4 × 10^7^ L mol^−1^) that includes a strong binding site and other weaker sites^44^. For instance, substituted aluminum (III) phthalocyanines interact with domains I and III in bovine serum albumin as the primary and secondary binding sites, respectively^45^. Taking into consideration these high affinity interactions, the design of phthalocyanine–albumin assemblies as novel nanosystems has been explored^46^. These interactions may also modulate the properties of albumin aggregation causing changes on the number of aggregations, which in turn, modulate NP size. Noteworthy, despite the discussed size increase, DDS capacity to reach the brain parenchyma and deliver dopamine or AlClPc was not affected.

Further investigation detected the presence of our traceable fluorescent NPs mainly at the striatum and hippocampal formation. Our findings are in line with earlier studies where fluorescent labeled PLGA nanoparticles were also detected at the hippocampal formation^32,47^ and at the striatum^12^. Remarkably, our ALNP + AlClPc were more abundant at the striatum, depicting a relevant finding considering that this region is highly affected in PD. Our analysis displayed that the enhanced penetrating albumin/PLGA nanosystem possesses physicochemical and biochemical characteristics that enabled it to ultimately reach the brain parenchyma by circumventing the highly selective and cohesive structure of the BBB^40,48,49^. Thus, considering size, negative surface charge, lipophilic nature, and structural composition, we elucidate that the crossing of the BBB endothelial cell layer possibly used transcellular routes, such as lipid-mediated diffusion or/and adsorptive transcytosis via albumin-binding protein-mediated actively targeting the brain^28,49,50^. Given that albumin was used as part of our nanosystem’s structure, we expected to enhance NP avidity, providing biological signals that interact with specific receptors/transporters expressed by endothelial cells^40^. Albumin aided nanoparticle bypass through the BBB possibly by endogenous albumin pathways^28,40^ involving gp60 receptor mediated transcytosis^36^, effectively delivering dopamine at the site of action.

Mice with Parkinson’s disease-like symptoms treated with our ALNP + DA 20 mg/animal remained on the rotarod for a longer time, showing significant improvement in motor coordination and balance as a result from delivery and responsiveness to therapeutic dopamine replacement, suggesting motor deficit recovery. Additionally, no significant difference was observed when contrasting Sham and ALNP + DA 20 mg rotational results, suggesting that our nanoparticle-based treatment at this specific dose restored animal performance to a level comparable to that seen in non-lesioned animals. As an approach to complement our neurobehavioral tests, the adhesive removal test provided additional sensitivity to detect subtle alterations in the nigrostriatal dopamine system and basal ganglia function^51^ through the response to sensory stimuli. Animals treated with ALNP + DA 20 mg presented similar results to Sham control group with respect to time to remove adhesive when contrasted to untreated 6-OHDA-lesioned animals. Consistent with the results obtained for the other behavioral tests, animals treated with our dopamine-loaded albumin/PLGA nanosystem (20 mg) bypassed the BBB, effectively delivering and replenishing the dopamine content at the brain tissue, restoring sensorimotor function (dexterity) performance comparable to that seen in healthy animals treated only with deionized water at the striatum and i.p. with saline.

Assessment of apomorphine-induced spontaneous rotations showed that mice treated with either dose of ALNP + DA presented significant reduction in the number of apomorphine-induced rotations when compared to untreated Lesioned animals. When apomorphine is administered to 6-OHDA-lesioned animals, a dopamine receptor agonist that acts post-synaptically causes hyperstimulation of supersensitive dopaminergic receptors in the denervated striatum, leading to rotations in contralateral circles^52,53^. Since apomorphine-induced rotations depend on dopaminergic receptor supersensitivity, our results suggest that this sensitivity is reduced as dopamine was delivered and released by our albumin/PLGA nanosystems, replenishing dopamine at the denervated dopaminergic nigrostriatal pathway, at a level congruent to that observed in Sham animals. On this aspect, Chen et al.^54^ showed that an increment in dopamine release and reuptake in 6-OHDA-lesioned mice submitted to exercise, reduced dopaminergic receptor supersensitivity, as observed in the apomorphine-induced rotation test that depends on this sensitivity. Overall, these results corroborate the relationship between apomorphine-induced rotation intensity and striatal dopamine content, considering that the extent of dopamine loss is a critical factor in determining the degree of developed supersensitivity^55^.

Altogether, our neurobehavioral test results indicated that dopamine carried by our albumin/PLGA nanosystems, even at its lowest dose, resulted in significant symptomatic improvements when compared to the untreated 6-OHDA-lesioned group and to the animals treated with bulk L-DOPA. In the case of the highest dose used (ALNP + DA 20 mg equivalent to 140 µg dopamine/animal), motor coordination, balance, and sensorimotor performance were restored to Sham level. A similar efficacy result was observed by Pahuja et al.^12^, who treated rats presenting Parkinson’s disease-like symptoms with dopamine-loaded nanoparticles and challenged these animals with parallel neurobehavioral tests. Nevertheless, in order to restore performance to Sham levels, animals needed a double infusion of NPs (293 µg dopamine total/animal), a two-fold dose in comparison to our most efficacious ALNP dose. Comparing both studies suggests that it is not only the encapsulation technique that is important, but also the structure of the nanoparticle. In this case, the incorporation of albumin to the nanosystems’ surface enhanced interaction with specific endothelial cell membrane receptors at the BBB, promoting the nanosystem’s passage, thus successfully reaching the site of action at the brain parenchyma.

With respect to the damage extent caused by 6-OHDA injection on DA neurons, along with the nanoparticles’s possible neuroprotective effect, TH^+^-immunolabeling of viable SN dopaminergic neurons revealed that independently from the treatment group, the percentage of neurons from the lesioned side was reduced when compared to DA neurons at SN from the non-lesioned side. This indicates that the proportion of dopaminergic neuronal death was similar in all groups, suggesting the absence of a neuroprotective effect for any of the treatments tested. Considering that our experimental treatment is based on the replacement of dopamine at the brain tissue by DDS, it was expected that the administration and consequent replacement of this neurotransmitter would result only in the clinical improvement of symptoms, having no effect on disease progression or dopaminergic neuronal death, consistent with earlier in vivo studies^12,23,24^.

## Conclusions

As evidenced in the current study, the specific physicochemical characteristics displayed by our albumin/PLGA nanosystems permitted successful BBB crossing, consequently allowing drug delivery and animal treatment. Our neurobehavioral results showed that dopamine encapsulated in albumin/PLGA nanosystems at the highest dose used displayed significant symptomatic improvements when compared to untreated animals with Parkinson’s disease-like symptoms and to group treated with bulk L-DOPA, currently used in patients as frontline treatment against PD. Most importantly, our dopamine-loaded-ALNPs restored motor coordination, balance, and sensorimotor performance to non-lesioned animal level. This suggests that ALNPs 20 mg successfully delivered, up-regulated, and replenished dopamine levels, ultimately leading to motor symptomatic improvement. On the other hand, groups treated with unloaded NANO and unloaded ALNPs showed no difference when contrasted with the 6-OHDA-lesioned group. Furthermore, dopamine encapsulated in the NANO-NPs did not demonstrate a significant improvement in the tests performed, indicating that it is not enough to be a "polymeric nano" to effectively permeate the BBB, but it is also necessary to involve an appropriate delivery system that signals BBB opening while preserving the pharmacological activity of the encapsulated drug. Thus, it is not only the encapsulation technique that is important, but also the structure of the whole nanosystem. In this case, the incorporation of albumin on the nanosystems’ surface enhanced the interaction with specific endothelial cell membrane receptors, promoting NP passage through the BBB, reaching the site of action. Taken together, the administration of dopamine-loaded albumin/PLGA nanosystems suggests a potentially innovative and efficacious drug delivery system for the symptomatic treatment of Parkinson’s disease. Furthermore, considering the efficacy of our nanoplatform to load and deliver even hydrophilic drugs and circumvent the BBB, we propose its use as a relevant and potential strategy intended for the treatment of brain tumors or other neurodegenerative diseases.

## Methods

### Nanoparticle preparation

The development of the nanoparticles used herein was accomplished at the Center for Nanotechnology and Tissue Engineering at the Faculty of Pharmaceutical Sciences, Ribeirão Preto, University of São Paulo (USP). Nanoparticles were synthesized under aseptic conditions following previous methodology^56,57^, with slight modifications. Basically, we worked with two types of nanoparticles: (a) PLGA polymeric nanoparticles (NANO); (b) NANO NPs associated to lipid and bovine serum albumin (BSA) NPs, thus forming a nanosystem, named ALNP. BSA was used to change the superficial behavior of the nanosystem’s core, allowing signalization for the BBB opening and NP crossing^28^. Each nanoparticle type had sub-variations for unloaded-control nanoparticles (NANO-CT and ALNP-CT) and dopamine delivery systems (NANO-DA and ALNP-DA). Batches of unloaded-ALNPs were additionally photolabed with fluorophore aluminum chloride phthalocyanine (AlClPc) (ALNP-AlClPc) for nanoparticle tracking across the BBB.

**Figure 10:**
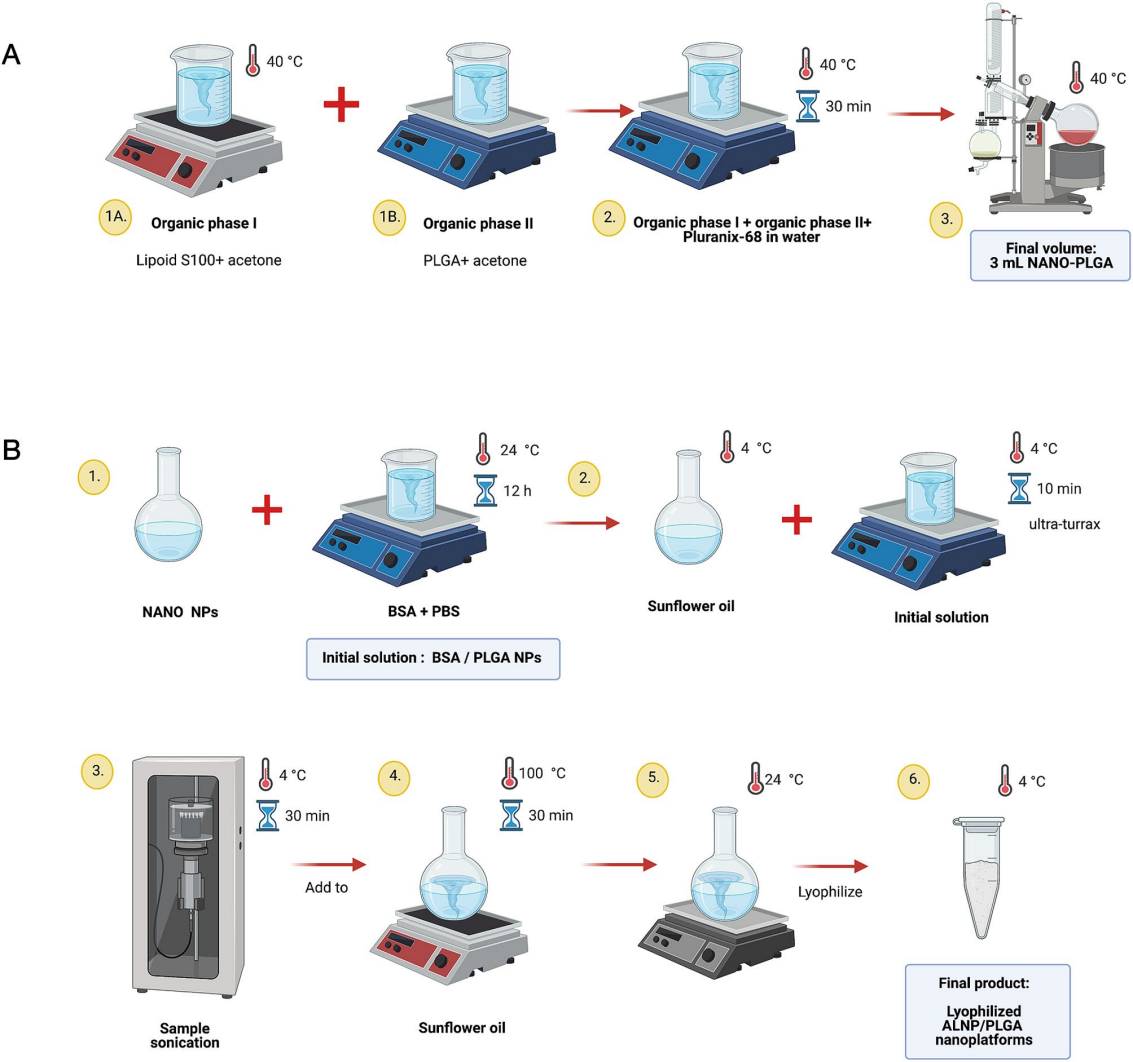
Simplified procedure used to synthesize (**A**) NANO-PLGA nanoparticles and (**B**) Albumin/PLGA nanosystems. PLGA:poly lactic-co-glycolic acid. Created using BioRender.

NANO nanoparticles were synthesized first by preparing an “organic phase I”, dissolving 175 mg of Lipoid S100 (Lipoid GMBH, Germany) in 10 mL of acetone HPLC grade (J.T. Baker^®^, USA). This mixture was placed under constant magnetic stirring, using a Corning^®^ stirrer/hot plate (Sigma-Aldrich Ltd., USA) at 870 rpm with a thermostatic bath at 40 °C. An “organic phase II” solution was prepared by adding 75 mg of 50:50 PLGA (Sigma-Aldrich Ltd., USA) in 5 mL of acetone. When aluminum chloride phthalocyanine was used in the formulation, 0.5 mg of AlClPc (Sigma-Aldrich Ltd., USA) were dissolved into 250 μL of soybean oil (Sigma-Aldrich Ltd., USA) (AlClPc real concentration: 1.3 µg/mg nanoparticles) and then added to the “organic phase II” under constant sonication (Branson 2210 Ultrasonic Cleaner, Branson Ultrasonics, USA) for 15 min. The same procedure was applied to incorporate dopamine (Sigma-Aldrich Ltd., USA) at 7 µg/mg nanoparticles (please see section entitled: “Determination of effective dopamine load carried by nanoparticles” for more details). After homogenization of both organic phases, the entire content was slowly added to a water jacket containing 100 mg of Pluranix-68 (Sigma-Aldrich Ltd., USA) (serving as an aqueous phase allowing mixture homogenization) kept for 30 min under constant magnetic stirring on a hot plate set at 40 °C. Once homogenized, this solution was placed in a round-bottomed flask for rotary evaporation (Rotavapor^®^ R-215, Buchi Labortechnik AG, Switzerland) using a bath set at 40 °C to obtain a final volume of 3 mL (Fig. 10A).

In the next step, each type of NANO PLGA nanoparticle sub-variation (NANO-CT, NANO-DA, NANO-AlClPc) was associated to a lipid and bovine serum albumin system to ultimately form the following sub-variations: ALNP-CT, ALNP-DA and ALNP-AlClPc. With this objective, aqueous solutions of BSA (250 mg/mL) were prepared in saline phosphate buffer (PBS) at pH = 7.4, then added to 250 μL of each sub-variation of NANO nanoparticle and kept under constant stirring for 12 h at room temperature, forming an initial solution. Simultaneously, 30 mL of sunflower oil (containing 1% of Span 80, v/v) (Sigma-Aldrich Ltd., USA) were cooled down to 4 °C and added to a boiling flask. The initial solution containing albumin/PLGA nanoparticles was added to this flask (at a rate of 6 drops per minute) under continuous stirring using the ultra-turrax setup (13,000 rpm) for 10 min with the system temperature fixed at 4 °C. Mixture was then emulsified by ultrasound using a sonicator for 30 min at 125 W and 4 °C. Using another boiling flask, 70 mL of sunflower oil (containing 1% of Span 80, v/v) were pre-heated at 100 °C under continuous agitation for 30 min. Thus, the emulsion containing albumin/PLGA/oil nanoparticles was gently added to the pre-heated sunflower oil/Span and kept under continuous agitation at moderate rates. Once emulsified, the entire system was stirred at 7000 rpm for 10 min at 100 °C. Final suspension was then cooled down to room temperature under continuous magnetic stirring, washed with ethyl ether (3 × 30 mL) for oil separation, followed by centrifugation at 10,000 rpm for 30 min. After washing the “pellet” containing the new nanoparticle batches, the solutions were lyophilized to remove any remaining water trace and stocked at 4 °C until use (Fig. 10B).

### Nanoparticle characterization

#### Particle size

Particle hydrodynamic diameter (HD) and polydispersion index (PdI) were measured by laser dynamic light scattering, and zeta potential (Zt) was measured by electrophoretic mobility. Nanoparticle suspension samples were analyzed in a Zetasizer Nano ZS90 (Malvern^®^ Instruments, UK) using a 1.0 cm quartz cell, at 25 °C, in the range of 100–2000 Hz. Values were determined as the mean of three runs (10 measurements/run). Furthermore, shape and size of our ALNP + AlClPc nanosystems were analyzed by transmission electron microscopy (TEM). Briefly, samples (diluted in 1:1000 in water) were disposed in a carbon film-coated copper grid, fixed and contrasted with 2 μL of phosphotungstic acid (PTA) 2% for 24 h in a semi-open Petri plate before analysis using a transmission electron microscope (JEM-2100, JEOL, Japan) with 200 kV acceleration voltage. Electronic microscopy analyses were performed at the Federal University of Goiás—Laboratory of High Resolution Microscopy (LabMic/ UFG).

### In vivo studies

In vivo studies were conducted to evaluate if the NPs were able to cross the BBB and to determine symptomatic treatment efficacy using the unilateral 6-hydroxydopamine (6-OHDA) PD mouse model that allows quantifiable lateralized behavioral tests.

#### Animals

In total, 120 adult Swiss males (*Mus musculus*) (ranging from 5 to 7 weeks of age and 20–35 g of body weight) were used. All animals were obtained from the animal facility at the Institute of Biological Sciences, University of Brasilia. Mice were kept under constant and controlled 12-h light/dark cycles, temperature (25 °C) and humidity (55%) and received water and food (standard pellets) ad libitum. Experiments were in compliance with NIH guidelines and ARRIVE guidelines, and were properly approved by the University of Brasilia Animal Ethics Committee (UnBDOC no. 63999/2011). All animal handling was conducted with proper humane care.

### Nanoparticle delivery across the BBB

Animals were randomly separated into three experimental groups, each group with *N* = 5–7, exposed according to the experimental setup:

- **Groups ALNP/AlClPc 10 mg and ALNP/AlClPc 20 mg** Mice received an intraperitoneal (i.p.) injection with 200 μL solution containing either 10 mg (0.05 mg/μL) or 20 mg (0.1 mg/μL) of albumin/PLGA nanoparticles (ALNP), functionalized with photoactive compound aluminum chloride phthalocyanine, diluted in saline solution.
- **Vehicle** Animals were injected i.p. with 200 μL of saline solution.

One hour after nanoparticle systemic administration, animals were euthanized using a CO_2_ chamber followed by cardiac perfusion in the left ventricle with 0.9% saline and 4% formaldehyde. Brains were manually removed from the animals’ skulls and fixed in 4% formaldehyde for 24 h. After this period, brains were cryopreserved by immersion in a 30% sucrose solution (0.1 M phosphate buffer, pH 7.4) for 48 h at 4 °C. Once fixed, a vibratome (KEDEE^®^, KD-400, Zhejiang Jinhua Kedi Instrumental, China) was used to section the brains into coronal slices (50 μm thickness) containing the striatal and hippocampus regions (including the *substantia nigra*), based on Paxinos and Franklin^58^. All slices were immediately placed in an anti-freezing solution (30% glycerine and 30% ethylene glycol) at 4 °C until further processing.

Brain sections were rinsed in PBS solution twice for 10 min for complete removal of the anti-freezing solution and incubated for 1 h in 0.3 M solution of glycine at room temperature under constant stirring. Slices were rinsed again with PBS, mounted on gelatinized slides, and labeled with DAPI (4′6′-diamidino-2-phenylindole) fluorescent dye marker (Sigma-Aldrich Ltd., Brazil) and left at room temperature before placing the coverslips and sealing with colorless enamel. Slides were imaged using an epifluorescence microscope (Leica^®^ model DM2000, Leica Microsystems, Switzerland) and digitally photographed using a camera (Leica DFC280) and software (Leica Application Suite Version 2.7.0 2003–2007, Leica Microsystems, Switzerland). Microphotographs were acquired at 10×, 20×, and 40× magnification using the blue, green, red filters, and an overlay of all filters. Analysis aimed to determine if the fluorescent nanoparticles were able to cross the BBB, cerebral localization, and quantification.

### Determination of effective dopamine load carried by nanoparticles

DA dose necessary to be entrapped in NPs to deliver effective symptomatic improvement was determined by performing a preliminary test with direct intracranial bulk dopamine (Dopacris^®^, injectable solution 5 mg/mL, Cristália, Brazil) infusion into the brain of 6-OHDA-lesioned animals. Both DA doses (35 and 70 µg/animal) tested were considered non-toxic based on an earlier study^12^. Mice were randomly divided into three experimental groups, *N* = 7 per group, an treated as follows:

- **Group lesioned** Unilateral 6-OHDA-lesioned mice treated i.p. with saline.
- **Group DA 35 µg/animal** Unilateral 6-OHDA-lesioned mice treated via intracerebrocentricular (i.c.v.) with bulk dopamine at a dose of 35 µg/animal.
- **Group DA 70 µg/animal** Unilateral 6-OHDA-lesioned mice treated i.c.v with bulk dopamine at a dose of 70 µg/animal.

Mice were anesthetized i.p. with ketamine (Dopalen, Ceva^®^, Brazil) and xylazine (Anasedan, Ceva^®^, Brazil) (75 and 15 mg/kg, respectively) diluted in saline (0.9%), and then placed in a stereotaxic frame (Insight Equipamentos^®^, Brazil). After proper asepsis and removal of head hair, a local subcutaneous injection of lidocaine 2% (Lidostesim^®^ 3%, Dentsply Pharmaceutical^®^, Brazil) was given prior to expose the skull, making a small incision in the scalp. A dental drill was used to make a small craniotomy at the following striatal coordinates: antero-posterior (AP): 0.0; medio-lateral (ML): − 2.5; dorso-ventral (DV): − 3.5 relative to bregma^37^. The nigro-striatal pathway was lesioned unilaterally by intracranial administration of 6-OHDA (Sigma-Aldrich Ltd., Brazil, 40 μg/animal of freebase) using a Hamilton syringe (Hamilton Company^®^, Nevada, USA) linked to an infusion pump (Harvard Apparatus Compact^®^, MA, USA) at a rate of 0.5 μL/min. A guide cannula (stainless steel 10 mm length and 0.7 mm of external diameter) was implanted in the right lateral ventricle at the following coordinates in relation to bregma: AP: + 0.2, L: + 1.0, V: − 2.3 mm^37^. After 4–6 days of recovery, bioassays were performed. Treatments were administered i.c.v. through the guide cannula using the Hamilton syringe linked to the infusion pump, immediately before rotarod test. Twenty-four hours prior to the 6-OHDA lesioning, animals were trained in a rotarod apparatus (Insight Equipamentos Ltda^®^, São Paulo, Brazil) for a total of five trials, with a resting interval of 5 min between each trial. In each training trial, animals were allowed to walk on the rod for 5 min, and only animals that successfully completed this task were then randomly allocated to the experimental groups. All animals were placed on the rod for three times and latency to fall was registered. Mice were challenged every 15, 30 and 60 min after dopamine administration.

### Neurobehavioral studies

#### Preparation of PD mouse model through 6-OHDA lesioning and subsequent treatment

Animals were randomly divided into experimental groups, *N* = 7 per group, an exposed as per the following experimental setup:

- **Group sham** Animals were injected with deionized water at the striatum (at the same coordinates as Group lesioned with 6-OHDA) and treated i.p. with saline.
- **Group lesioned** Unilateral 6-OHDA-lesioned mice treated i.p. with saline.
- **Group L-DOPA** Unilateral 6-OHDA-lesioned mice received intravenous (i.v.) infusions of bulk L-DOPA/benserazide i.p. (6 mg/kg and 5 mg/kg).
- **Group NANO + dopamine** Unilateral 6-OHDA-lesioned mice treated with single i.p. infusion of dopamine (7 µg/mg nanoparticle) encapsulated in PLGA nanoparticles (NANO + DA), used as control, in two different doses: 10 mg/animal and 20 mg/animal.
- **Group ALNP + dopamine** Unilateral 6-OHDA-lesioned mice treated with single i.p. infusion of dopamine (7 µg/mg nanoparticle) encapsulated in albumin/PLGA nanoparticles (ALNP + DA), i.p., in two different doses: 10 mg/animal and 20 mg/animal.
- **Group NANO-CT** Unilateral 6-OHDA-lesioned mice treated with single i.p. unloaded PLGA nanoparticles (NANO), in two different doses: 10 mg/animal and 20 mg/animal.
- **Group ALNP-CT** Unilateral 6-OHDA-lesioned mice received single i.p.infusion of/unloaded albumin/PLGA nanosystems (ALNP), i.p., in two different doses: 10 mg/animal and 20 mg/animal.

In brief, animals were anesthetized as described in the “Determination of effective dopamine load carried by nanoparticles” section*.* For the injection of vehicle solution (saline) or 6-OHDA, a dental drill was used to make a small craniotomy above the left striatum region according to the following stereotaxic coordinates in relation to bregma: AP: + 0.98 mm, ML: − 1.5 mm, DV: − 3.5 mm^58^. A volume of 4 μL of saline or 6-OHDA (Sigma-Aldrich Ltd., Brazil, 40 μg/animal of freebase) were intracranially injected using a Hamilton syringe (Hamilton Company^®^, Nevada, USA) linked to an infusion pump (Harvard Apparatus Compact^®^, MA, USA) at a rate of 0.5 μL/min. Animals injected with 6-OHDA resulted in hemiparkinsonian mice with selective loss of nigrostriatal dopaminergic neurons^59^. Once the surgical procedure was completed, skulls were covered with dental acrylic. Throughout the experimental period, animals were fed daily with softened food pellets and water.

Once the 6-OHDA lesioning was performed, two treatment protocols were applied to compare the antiparkinsonian effect in animals treated with dopamine-loaded nanoparticles and bulk L-DOPA, used as the positive control. Noteworthy, both protocols had the same experimental duration, time points, and order for sensorimotor battery testing. Nevertheless, animals from the nanoparticle group were treated only once, while animals in the L-DOPA group were treated in five different time points for a total of nine treatment doses. Sensorimotor tests were then conducted and consisted of the rotarod test, the adhesive removal test, and the apomorphine-induced rotation tests.

### Rotarod test

This test is widely used to assess motor coordination and balance in mice^60^. Twenty-four hours prior to the 6-OHDA lesioning, animals were trained in the rotarod for a total of five trials, with a resting interval of 5 min between each trial. In each training trial, animals were allowed to walk on the rod for 5 min, and only animals that successfully completed this task were then randomly allocated to the experimental groups. For the actual test, which occurred on experimental days 4 and 5 (24 h and 48 h post-treatment administration), all animals were placed on the rod only once and latency to fall was registered. In the case of animals treated with L-DOPA/benserazide, tests were performed on experimental days 4 and 5, 30 min after drug administration.

Latency to fall was also recorded. In this case, all animals were tested on experimental day 3 by being repeatedly placed on the rod, on the following time points: 15, 30, 45, 60, 90, 120, 150, 180, 210, 240, 300, and 360 min, for a total of 6 h. Moreover, 24 h and 48 h post-treatment administration (1440 and 2880 min), all animals were placed on the rod only once and latency to fall was registered. In the case of animals treated with L-DOPA/benserazide, tests were performed on experimental days 4 and 5, 30 min after drug administration.

### Adhesive removal test

This task is carried out to assess fine motor skills, measuring the animals’ ability to detect a stimulus, such as a small adhesive sticker placed on the forepaw and the ability to remove it. Animals used in PD models show an increase in the time required to remove this sticker^60^. Task was performed in all animals before 6-OHDA lesioning and on experimental day 7. Prior to surgery, training was performed to acclimate the mice to the sensory stimuli (adhesive tape measuring 0.8 × 0.8 mm). On experimental day 7, after 6-OHDA or saline injections, adhesive tape was placed on the right forepaw (contralateral to the lesion). The time to complete this task (latency) was recorded using a stopwatch, having a maximum of 300 *s* to reach the objective (adapted^61^). Median data were calculated across three independent trials.

### Apomorphine-induced rotation tests

This test is applied to assess locomotor activity, specifically motor asymmetry, in rodents with unilateral 6-OHDA lesions that cause a rotational bias toward the side of the lesion, altered by drug treatments^60^. On the last experimental day, animals received a subcutaneous injection of apomorphine (0.5 mg/kg body weight diluted in NaCl saline solution 0.9%), a dopaminergic agonist. Animals were placed in an arena and 30 min post-injection, they were filmed for 5 min and the number of contralateral rotations was manually recorded.

Once the behavioral tests were completed, animals were euthanized using a CO_2_ chamber followed by cardiac perfusion in the left ventricle with 0.9% saline and 4% formaldehyde. Brains were manually removed from the skull and fixed in 4% formaldehyde for 24 h and then placed in 30% sucrose for 48 h. Once fixed, the brains were sectioned into coronal slices (50 μm each) using a vibratome (KEDEE^*®*^, KD-400, Zhejiang Jinhua Kedi Instrumental, China). Additional slices were also prepared to certify that the 6-OHDA lesioning was correctly done at the striatum. All slices were immediately stocked in an anti-freezing solution (30% glycerine and 30% ethylene glycol at 4 °C) until further processing.

### TH^+^-immunohistochemistry

Immunohistochemical staining of brain tissue sections was performed using antibody tyrosine hydroxylase (TH^+^), a useful marker for dopaminergic neurons. For this, brain slices were removed from the anti-freezing solution and rinsed twice with PBS for 10 min in constant agitation. Slices were then permeabilized in PBS containing 0.8% Triton X-100 (Sigma-Aldrich, Brazil) for 1 h. Samples were rinsed five times for 5 min under agitation in PBS and then incubated in protein block (1% BSA, 10% skimmed milk, 0.3 M glycine, and 0.1% Tween 20) for 1 h. After blocking, samples were rinsed three times with PBS for five minutes and incubated in primary anti-body for 48 h at 4 °C under constant stirring. Samples were washed with PBS three times for 1 min each and then incubated with fluorescent secondary antibody for 3 h at room temperature, in the dark, under stirring. Slices were then rinsed three times for 4 min in the dark. Sections were then mounted on glass slides and cell nuclei were stained with DAPI, let to dry, and covered with slips. Slides were imaged using an epifluorescence microscope (Leica^*®*^ model DM2000, Leica Microsystems, Switzerland) and digitally photographed using a camera (Leica DFC280) and software (Leica Application Suite Version 2.7.0 2003–2007, Leica Microsystems, Switzerland). Analysis aimed to count the number of neurons reactive to TH^+^ at the *substantia nigra* and at the Ventral Tegmental Area (VTA) in both hemispheres (left ipsilateral: 6-OHDA lesion, right contralateral: non-lesioned). The following formula was used to calculate the % of remaining TH^+^ neurons:

$$\frac{{\text{quantity of neurons at the ipsilateral side}}}{{\text{quantity of neurons at the contralateral side}}} \times 100$$

### Statistical analysis

Analysis were carried out by using GraphPad Prism statistical analysis software version 8.0 (GraphPad^*®*^, La Jolla, California, USA). Normality tests (Shapiro–Wilk, Kolmogorov–Smirnov, D’Agostino and Pearson) were adopted to determine if data were normally distributed prior to statistical testing. Parametric data were evaluated through one-way analysis of variance (ANOVA) followed by the Tukey post hoc multiple comparisons test. In the case of the non-parametric data, Kruskal–Wallis statistic was used, followed by Dunn as post-test. For the rotarod test, before performing the ANOVA, data were normalized using a Box–Cox transformation, which in this case was λ = − 0.4. Area under the curve (AUC) was calculated as an estimate of the total time each animal remained on the rotarod. AUC was used to eliminate any possible pseudo-replication (while running one-way ANOVA) by the trapezoidal method, considering that the same animal was tested at different times. Values of *p* < 0.05 were considered to be statistically significant.
